# Iterative Reconstruction of High-Dimensional Gaussian Graphical Models Based on a New Method to Estimate Partial Correlations under Constraints

**DOI:** 10.1371/journal.pone.0060536

**Published:** 2013-04-11

**Authors:** Vincent Guillemot, Andreas Bender, Anne-Laure Boulesteix

**Affiliations:** Department of Medical Informatics, Biometry and Epidemiology of the Faculty of Medicine, University of Munich, Munich, Germany; University of Catania, Italy

## Abstract

In the context of Gaussian Graphical Models (GGMs) with high-dimensional small sample data, we present a simple procedure, called PACOSE – standing for PArtial COrrelation SElection – to estimate partial correlations under the constraint that some of them are strictly zero. This method can also be extended to covariance selection. If the goal is to estimate a GGM, our new procedure can be applied to re-estimate the partial correlations after a first graph has been estimated in the hope to improve the estimation of non-zero coefficients. This iterated version of PACOSE is called iPACOSE. In a simulation study, we compare PACOSE to existing methods and show that the re-estimated partial correlation coefficients may be closer to the real values in important cases. Plus, we show on simulated and real data that iPACOSE shows very interesting properties with regards to sensitivity, positive predictive value and stability.

## Introduction

The robust estimation of the inverse covariance matrix is crucial in many multivariate statistical methods such as discriminant analysis or linear regression [1]. Many variants of these multivariate methods aim at somehow “regularizing” the estimation of the covariance matrix to make it invertible or better conditioned, *e.g.* ridge regression (RR), diagonal discriminant analysis or regularized discriminant analysis [2]. A large body of literature is devoted to the estimation of the inverse covariance matrix in high-dimensional small sample settings, *i.e.* when the number of observations 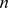 is much smaller than the number of variables 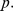 A well-known example is the shrinkage estimator by Shäfer & Strimmer [3] which is defined as a weighted sum of the sample covariance matrix and a fixed (invertible) target matrix. This method can be considered as “agnostic” in the sense that it estimates the covariance matrix in a completely data-driven way, *i.e.* without prior knowledge.

In this article, we first propose a method that directly estimates the partial correlation matrix while taking into account prior information on the dependencies between variables materialized by a given undirected graph. In a nutshell, our new method takes such a graph – called “independence graph” – as input and estimates the non-zero coefficients of the partial correlation matrix by regularized linear regression using the regression-based definition of partial correlation. The inverse covariance matrix can then be simply obtained from the partial correlation matrix by incorporating estimates of the variances. In this sense, our method can be seen as a *covariance selection* algorithm [4]. Although many covariance selection methods have been proposed in the literature (see below for details), none of these methods is designed to estimate the partial correlation matrix in high-dimensional settings while incorporating a *non-decomposable* independence graph. In reference to covariance selection, we called this first method “PACOSE', standing for **PA**rtial **CO**rrelation **SE**lection.

Furthermore, we suggest a new iterative algorithm called “iPACOSE” – standing for iterative PACOSE – that estimates an independence graph from a dataset using our new partial correlation estimate in a recursive way. Briefly, iPACOSE takes as inputs a dataset and a significance level for the partial correlation and gives as an output an estimated independence graph. We show on simulated datasets that recursive reestimation of the partial correlation coefficients yields graphs closer to the true graph than a simple thresholding of an estimated partial correlation matrix.

The rest of the paper is structured as follows. We first present our iterative method and the associated covariance selection and also briefly reviews existing covariance selection methods. Then, we compare our new method to existing estimation algorithms for Gaussian Graphical Models (GGM) on simulated data. Finally, we apply our method to real datasets.

For the sake of reproducibility, we made our code available in the form of:

- An R package called pacose, available on the CRAN http://cran.r-project.org/web/packages/pacose/index.html (Accessed 2013 March 13),
- A set of R programs for the reproduction of our results, available online at http://www.ibe.med.uni-muenchen.de/organisation/mitarbeiter/020_professuren/boulesteix/pacose2012/(Accessed 2013 March 13).

## Methods

### Context

The estimation of networks is a burning issue in bioinformatics. Gaussian graphical models (GGMs) [5], [6] have been widely used for this purpose in the last few years [3], [7]. In the context of systems biology, the estimation of GGMs is very often characterized by a lower number of individuals 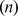 or measures than the number of variables 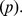 In this 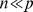 situation, regularization techniques are mandatory to enable the estimation of GGMs.

The core method of the present work is designed to estimate a partial correlation matrix under the constraint that some known coefficients are equal to zero. It is intimately related to so-called *covariance selection* methods, which can themselves be seen as methods able to estimate the covariance matrix or its inverse, the so-called precision matrix, (i) under the constraint that some coefficients in the precision matrix are null [4] or (ii) under the constraint that a certain amount of coefficients are equal to zero in the precision matrix [8], [9]. To avoid any confusion with these sensibly different definitions, we chose an acronym closely related to the parameters that we want to estimate: the partial correlations, hence the name of this core method: PACOSE, “PArtial COrrelation SElection”. The theory behind PACOSE is further described in the section “PACOSE”.

**Figure 1:**
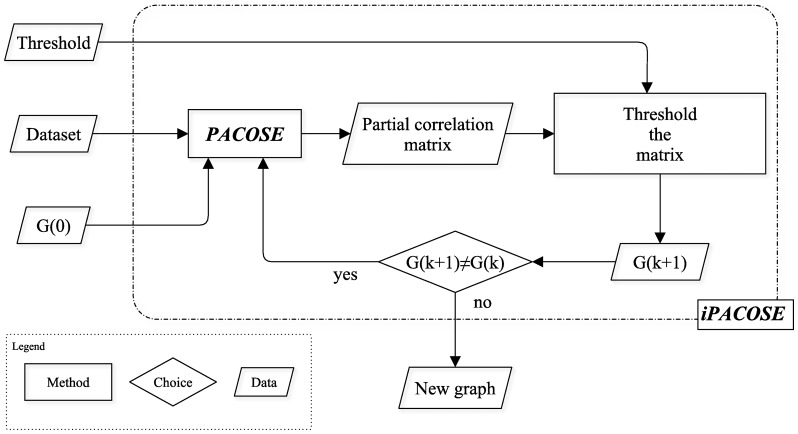
Flowchart representation of the iPACOSE algorithm, representing how it iteratively uses PACOSE to estimate an independence graph from a dataset.

We propose to embed PACOSE into an iterative algorithm designed to estimate independence graphs. The algorithm – called iPACOSE (standing for *iterative* PACOSE) – takes a dataset and a significance level for the partial correlation coefficients as inputs. PACOSE is then applied iteratively to the dataset to estimate an independence graph extracted from the previous iteration's partial correlation matrix by thresholding it. The iPACOSE algorithm is schematically represented in Figure 1. iPACOSE is described in more details in the section “iPACOSE”.

### Partial correlation and Gaussian Graphical Models

This section briefly reviews the basics of GGM theory used in this paper. Let 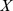 denote a 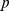-variate random vector 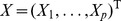 such that variables 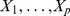 all have a mean and a variance. 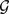 denotes the graph describing the conditional independencies between the 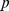 variables: 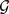 is thus an undirected graph with 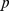 nodes. The covariance matrix of 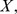 denoted by 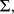 is supposed to be invertible. Its inverse 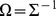 is from now on referred to as the *precision matrix*.

The partial correlation coefficient 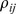 of 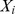 and 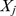 given all the other variables 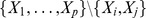 can be estimated as
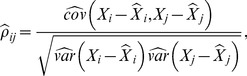
(1)where 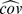 and 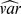 denote the empirical covariance and variance, respectively, and 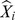 stands for the fitted value of 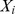 in a linear regression model including all other variables except 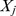 as covariates. In a few words, 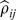 is the correlation of the residuals of the linear models regressing 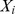 against all variables except 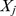 and vice-versa.

Another method to compute 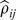 based on linear regressions results from the following property [6]:
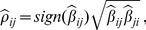
(2)where 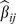 is the estimated coefficient of variable 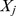 in the linear model regressing 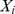 against all the other variables. Note that both formulations (1) and (2) implicitly assume that the considered linear regression models can be estimated, which is for instance not the case in high-dimensional data with 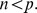 This issue will be discussed later. Moreover, it can also be shown [6] that the partial correlation coefficient 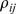 is related to the precision matrix 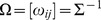 as follows:



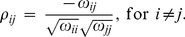
(3)If 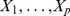 are Gaussian, the following important property can be shown for 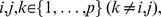, see for instance [10]:
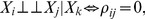
(4)which means that two variables are conditionally independent if and only if their partial correlation equals zero.

The formulation (4) is exploited by numerous methods to estimate gene regulatory networks from high-dimensional microarray gene expression data [7], [11], [12]. Note, however, that these data often have much more variables (genes) than observations (arrays), hence the term high-dimensional data”. A regularized regression technique has then to be used to estimate 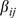 and 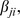 since least squares regression cannot be performed with 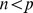 data. Another popular approach [3] to estimate GGMs from high-dimensional data consists in applying Eq. (3) using a regularized (invertible) estimator of 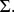


All these methods yield an estimate of the partial correlation matrix. Some methods are essentially sparse, *i.e.* yield a matrix with many zeros [11]. In this case, the graph is simply derived from the partial correlation matrix by connecting pairs of variables with non-zero partial correlations. For other methods [3], [7], however, a threshold has to be applied to decide which variables have to be connected.

### PACOSE

The concepts briefly reviewed in the above section are important for understanding our novel method – PACOSE -, whose main idea is to combine formulation (2) along with the information given in an a priori independence graph 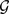 between the variables. This is done by setting 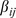 and 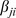 to 0 if 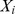 and 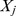 are not connected in the graph 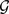. It immediately results from Eq. (2) that 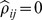.

Setting 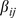 to 0 impacts the whole linear model
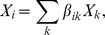
since it essentially removes one covariate in the regression model. As a consequence, the estimation of other partial correlation coefficients 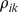 involving 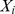 and any other variable 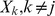 is also affected.

More precisely, our graph-constrained estimator of the partial correlation between 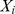 and 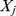 is given as
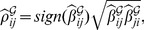
(5)where

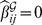
 if 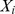 and 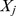 are not connected in 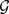,

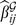
 is the estimated regression coefficient of 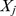 in the regression of 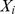 against its connected variables if 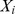 and 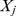 are connected, *i.e.* the estimate of coefficient 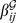 in the linear regression model
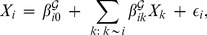
(6)where 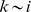 means that variables 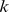 and 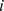 are connected in 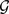.

This definition implicitly assumes that the estimates of the regression coefficients exist, which may not be the case in high-dimensional settings. This problem is addressed in the next section.

### High dimensional settings

When the number of variables connected to 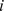 is greater than the number of observations, the estimation of the coefficients of the linear regression model (6) cannot be performed by ordinary least squares. Unfortunately, it is likely to sometimes occur in practical analyses with high-dimensional data. That is why we suggest to replace least squares regression by one of its regularized versions: ridge regression [13], PLS regression [14], [15], Lasso [16] or adaptive Lasso [17]. The regularization parameters are estimated by 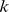-fold cross-validation (CV). Once the partial correlation coefficients are estimated, an estimator of the partial correlation matrix 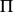 is obtained via Eq. (2).

### Competing approaches

To our knowledge, there is no method in the literature allowing to compute directly the partial correlation matrix with the knowledge of an undirected graph. But there are numerous methods dedicated to the estimation of the inverse covariance matrix knowing a given graph. The literature refers to these methods as covariance selection algorithms. These algorithms are usually used to estimate the covariance matrix, but they can also be used to estimate the precision matrix.

When the graph is decomposable, the covariance matrix can be estimated by maximum likelihood. Alternative methods have been proposed such as the shrinkage estimator designed by Wiesel *et al.* [10]. However, these methods are not able to cope with a non-decomposable graph. This is a major drawback in practice because most of the graphs relevant to bioinformatics are non-decomposable. One thus has to turn to iterative methods [6], [18] or methods such as “glasso” [11] based on the optimization of a criterion independently from the nature of the graph.

All the covariance selection methods we refer to in this section compute directly the precision matrix, and not the partial correlation matrix as PACOSE does. In order to compare PACOSE to them, we use Eq. (3) to transform any estimated precision matrix into a partial correlation matrix.

### iPACOSE

When estimating an independence graph from raw data with partial correlation matrices, one usually first estimates the partial correlation matrix and then applies to it a certain threshold, allowing to eliminate small coefficients. The obtained sparse matrix is then considered as the adjacency matrix of the underlying graph, following the principle of GGM.

The idea of the iPACOSE (as in “iterated PArtial COrrelation SElection”) algorithm is the following: rather than stopping after this first estimation of the underlying graph, we use this graph as an input for PACOSE, then allowing a re-estimation of the partial correlation coefficients. Since the newly estimated coefficients are likely to become smaller than the given threshold, a new graph can be estimated from this new partial correlation matrix by thresholding it, and so on. With this iterated process, we aim to estimate the coefficients close to the threshold more accurately and then eliminate as many false positive edges as possible.

More precisely, our algorithm iPACOSE takes a data matrix, a threshold and a graph (called 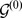) as inputs and operates as follows:

1. Apply PACOSE with the dataset and 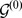 as arguments.
2. Transform the estimated partial correlation matrix into a graph by applying the threshold.
3. Apply PACOSE to the dataset with the graph derived in 2 in order to estimate a new partial correlation matrix.
4. Iterate steps 2 and 3 until the graph does not change anymore.

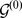
 can be estimated with any existing method, such as pcor.shrink from the R package GeneNet [3] or ridge.net, pls.net, adalasso.net or lasso.net from the R package parcor [7].

## Results

In this section we present a simulation study for the evaluation of

- PACOSE as an estimation procedure for the partial correlation matrix given a fixed undirected graph,
- iPACOSE as a procedure for graph estimation, in combination with standard GGM estimation procedures.

When one wants to simulate data knowing a given graph of independence, there is the possibility of using the theory of GGM, more particularly through the constraint (4). Furthermore, the randomly generated precision matrix has to be positive definite. One could see this problem as a so-called “positive definite completion matrix” issue [19]. But the work on this specific issue is once again mainly focused on decomposable graphs. We adopt a more empirical method, which in practice gives a very satisfying range of partial correlation coefficients, and at the end of the algorithm, the fulfillment of constraint (4).

### Simulated data

We use simulated data to compare our method to the methods presented in the literature. Erdös-Rényi [20] or Barabasi [21] graphs are used to model the interactions between genes, which allows loops, hubs, and multiple connected components. We use the following algorithm:

- Compute a first random Erdös-Rényi [20] (if we want a non-decomposable graph) or Barabasi [21] (if we want a decomposable graph) graph 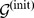,
- Get the “upper triangular” adjacency matrix 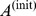 of this graph and replace any non null coefficient by a random realization of a uniform variable (*e.g.* 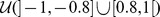, but any interval is possible), which then allows to define an upper triangular weight matrix 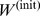,
- Compute the following matrix 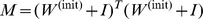, where 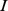 is the identity matrix, defining a new graph 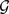 slightly different from the initial graph, but above all defining a sparse positive definite matrix 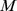,
- Normalize this matrix to get a partial correlation matrix 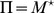,
- Generate the dataset from the multivariate Gaussian distribution 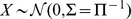.

We prefer this algorithm to *e.g.* the algorithm presented in Verzelen *et al.* [22] and Krämer *et al.* [7] because the latter produces partial correlation coefficients often very close to 0 when 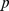 is greater than a few dozens. The drawback of this method is that it alters the degree distribution of the initial graph structure – in a drastic way for Erdös-Rényi graphs, and in a very moderate way for Barabasi graphs.

We implemented the covariance selection algorithm presented in [18] in R and C, and the minimum variance unbiased estimator (MVUE) and the Stein unbiased risk estimator (SURE) [10] in R. Whittaker's method [6] is implemented in the R package ggm, and Friedman's *et al.* method [11] in the package glasso.

### Estimation of the partial correlation matrix with PACOSE

We compare PACOSE to the competing methods presented above based on the mean square error (MSE) between the estimated partial correlation matrix (denoted 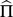) and the real one (denoted 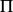), as defined by
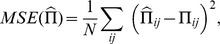
with 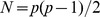.

The following notations are used for the competing approaches: GeneNet [3], glasso [11], MVUE, SURE [10], Whittaker [6], wermuth [18]. It has to be noted that GeneNet does not take into account the information in the given graph: it is considered in our results as a reference method giving an upper bound on the MSE.

These methods are compared to the PACOSE algorithm, where four different regularized regression methods are used to estimate the coefficients in Eq. (2):

- Ridge regression ( PACOSE(1)),
- PLS regression ( PACOSE(2)),
- LASSO regression ( PACOSE(3)),
- adaptive LASSO regression ( PACOSE(4)).

All the regularization parameters are estimated with 10-fold cross-validation. The graph used within PACOSE is the real independence graph, which is known since we work on simulated data.

**Figure 2:**
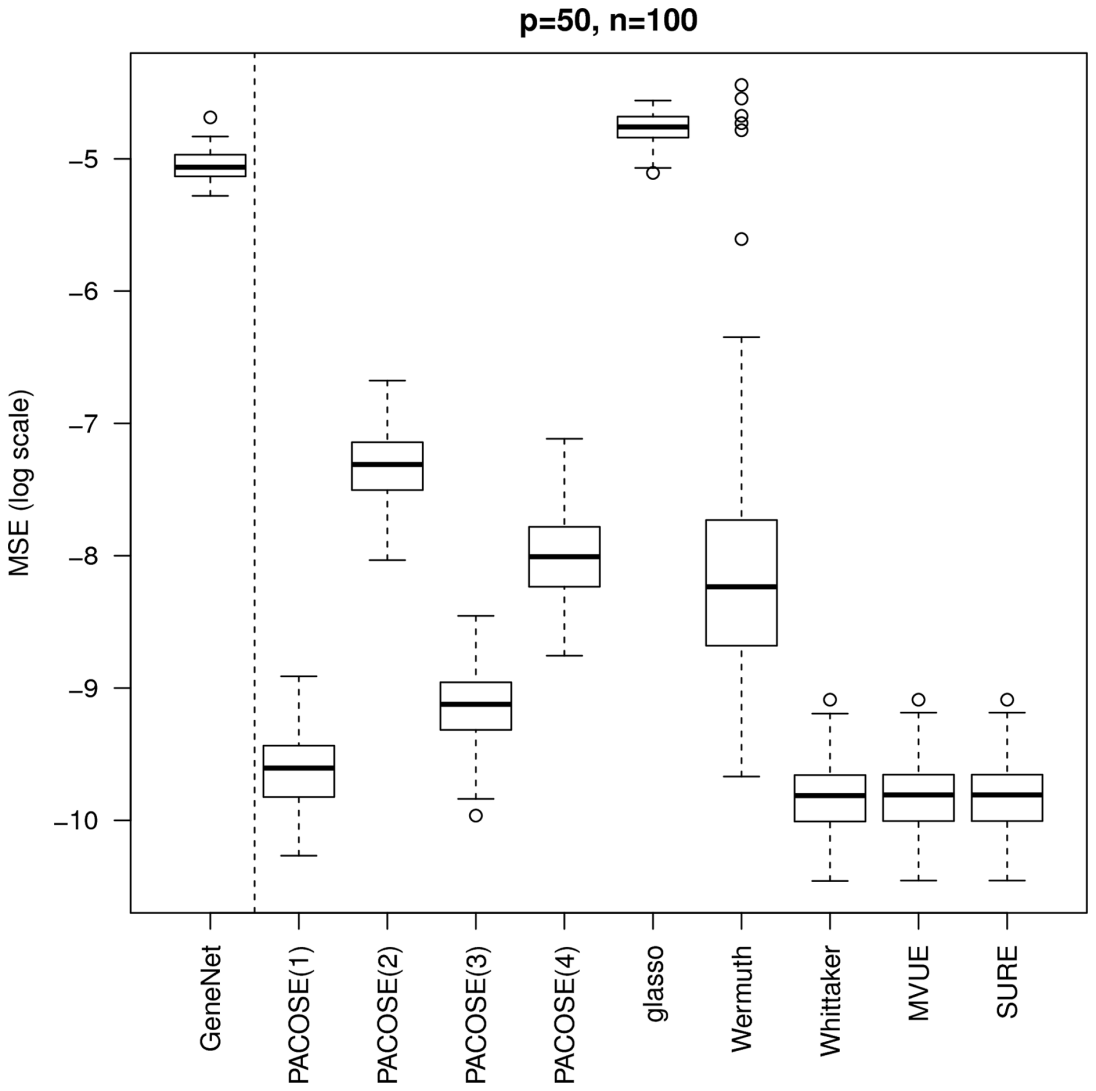
MSE of the partial correlation matrix estimates. 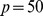
 and 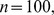 when the graphs are decomposable.

When there are more individuals than variables, and when the considered graphs are decomposable, we can see on Figure 2 that the SURE estimator performs better than all the others methods.

**Figure 3:**
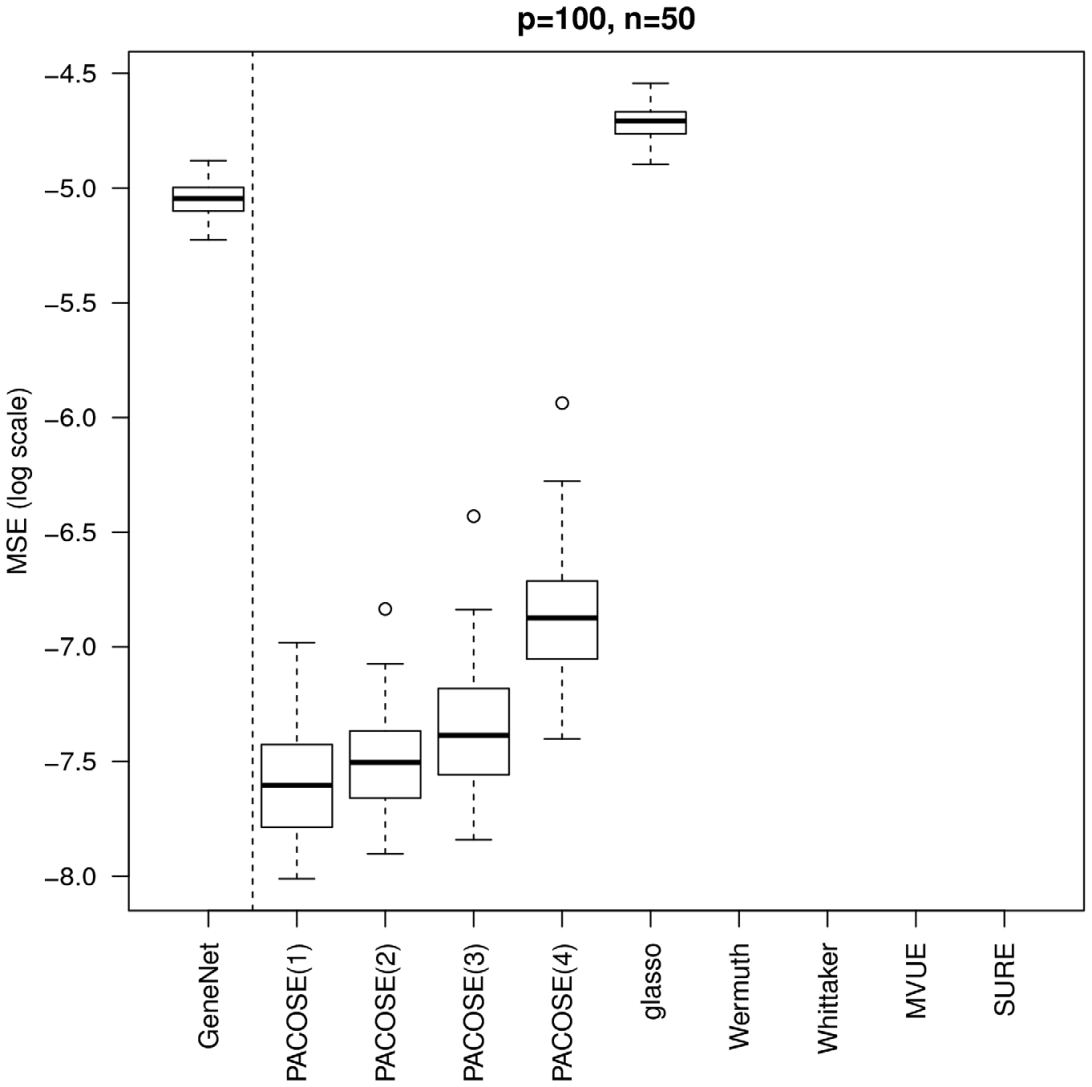
MSE of the partial correlation matrix estimates. 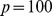
 and 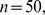 when the graphs are not decomposable. Since the graphs are not decomposable, the estimators MVUE and SURE are not applicable. Wermuth's algorithm does not converge, and the implementation of Whittaker's method requires a decomposition of the graph into cliques.

When the setting is less favorable, *i.e.* when there are less individuals than variables and the graphs are not decomposable, the results show a better performance of our estimator, both in terms of stability and accuracy, see Figure 3, especially for the PLS and Ridge regressions. This is a very promising result for PACOSE, since in reality the considered graphs are very unlikely to be decomposable, and the number of variables is generally bigger than the number of individuals. In both Figures 2 and 3, method GeneNet performs poorly, which is due to the fact that it does not consider the underlying graph. This method acts as a baseline representing the methods estimating the partial correlation matrix without any prior knowledge.

The underlying graphs of independence are not precisely known for biological data, estimating them being even a burning issue in bioinformatics. We show in the following that PACOSE can be advantageously integrated into the estimation of GGMs, yielding potential improvements in terms of estimation accuracy.

### Estimation of independence graphs with iPACOSE

In this section, we apply iPACOSE to simulated datasets in order to recover partial independence graphs. Our goal is to compare the four different network inference methods: ridge.net, pls.net, the non-adaptive version of adalasso.net and the adaptive version of adalasso.net based on the estimation of the partial correlation matrix, to their iterative versions iPACOSE(1), (2), (3), (4), respectively.

To compare the estimated graphs with the real graph, we use the positive predictive value (PPV, denoted 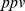) and the sensitivity (denoted 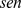):
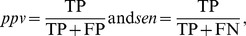
where TP, FP and FN are defined in Table 1. Biological networks are indeed often described as sparse, and indicators based on the number of edges are more suitable in this case [23].

**Table 1:** Prediction nomenclature in the context of graph inference.

	*i∼j*	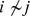
*p_ij_*≠0	TP	FP
*p_ij_*≠0	FN	TN

The definitions of true and false positives (resp. TP and FP), true and false negatives (resp. TN and FN) in the context of graph inference.

**Figure 4:**
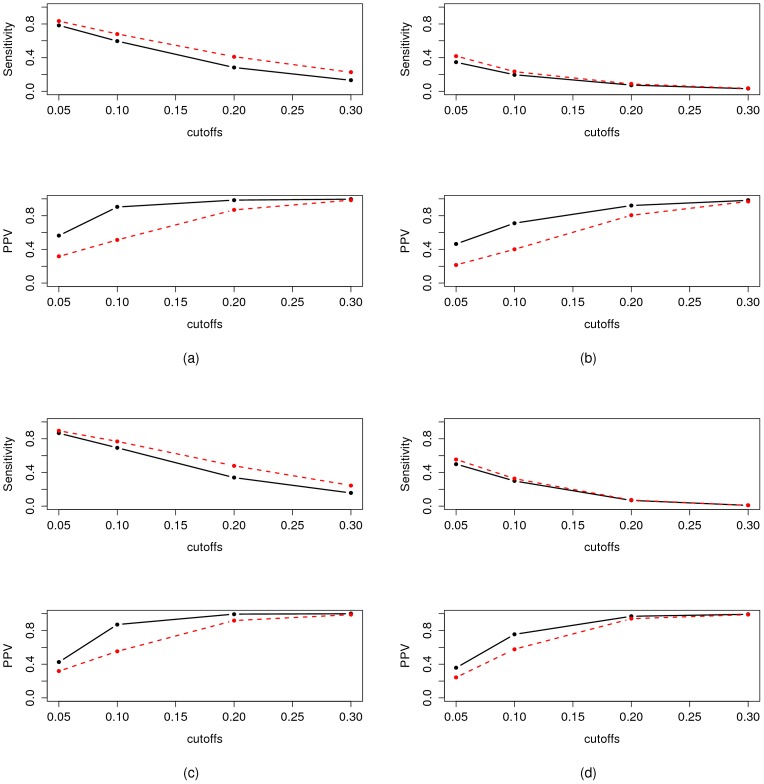
Performance of iPACOSE (black straight lines) when compared to its regression based GGM estimate counterpart (red dashed lines). *(a) and (b)*: performance of the PLS version of iPACOSE. 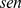 and 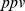 for 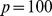 and 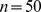 (a) and for 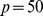 and 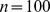 (b). Thresholds: 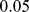, 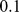, 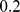 and 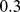. The results of iPACOSE are represented by the black line and the results of the pls.net function with the red dashed line. *UPPER FIGURE*: sensitivity as a function of the threshold, *LOWER FIGURE*: PPV as a function of the threshold. *(c) and (d)*: performance of the Ridge version of iPACOSE. 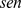 and 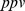 for 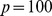 and 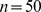 (c) and for 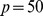 and 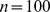 (d). Thresholds: 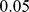, 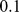, 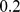 and 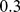. The results of iPACOSE are represented by the black line and the results of the ridge.net function with the red dashed line. *UPPER FIGURE*: sensitivity as a function of the threshold, *LOWER FIGURE*: PPV as a function of the threshold.

**Figure 5:**
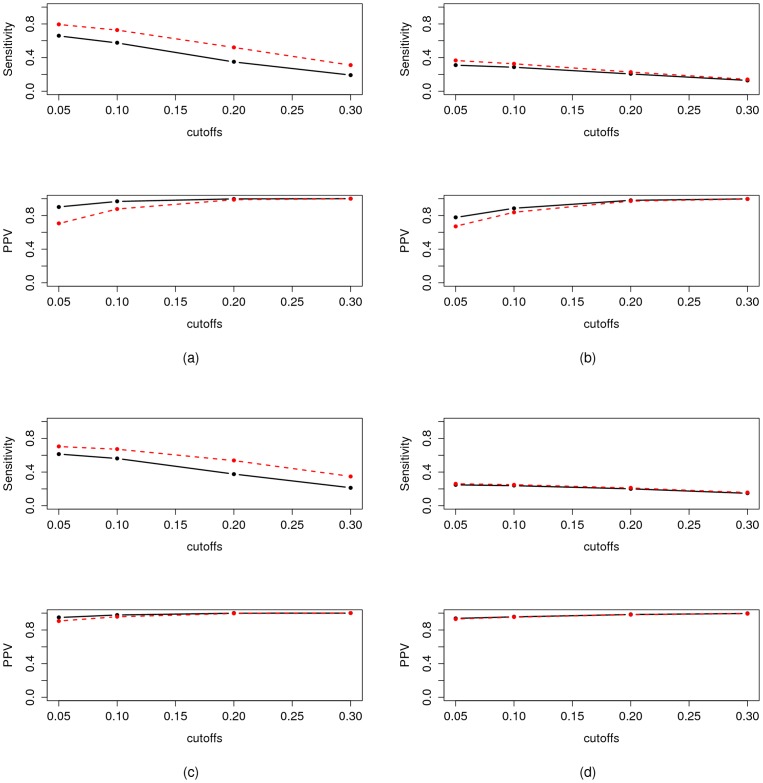
Performance of iPACOSE (black straight lines) when compared to its regression based GGM estimate counterpart (red dashed lines). *(a) and (b)*: performance of the LASSO version of iPACOSE. 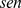 and 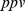 for 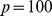 and 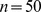 (a) and for 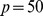 and 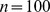 (b). Thresholds: 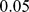, 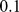, 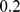 and 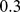. The results of iPACOSE are represented by the black line and the results of the adalasso.net function with the red dashed line. *UPPER FIGURE*: sensitivity as a function of the threshold, *LOWER FIGURE*: PPV as a function of the threshold. *(c) and (d)*: performance of the adaptive LASSO version of iPACOSE. 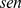 and 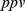 for 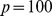 and 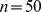 (c) and for 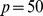 and 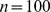 (d). Thresholds: 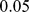, 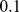, 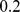 and 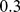. The results of iPACOSE are represented by the black line and the results of the adalasso.net function with the red dashed line. *UPPER FIGURE*: sensitivity as a function of the threshold, *LOWER FIGURE*: PPV as a function of the threshold.

The sensitivity and the PPV of the estimated graphs as a function of the threshold are represented on Figures 4 and 5. Two different settings are considered for these simulations: 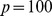 and 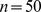 for Figures 4(a), 4(c), 5(a), 5(c), and 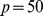 and 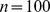 for Figures 4(b), 4(d), 5(b), 5(d). The key chacteristic of iPACOSE is that it allows to estimate networks with less edges without eliminating too many correct interactions. We can indeed observe on Figures 4(a)–(d) that the PPV, *i.e.* the capacity to estimate sparse networks, is improved when compared to ridge.net or pls.net. On the other hand, when applied to adalasso.net, a method estimating particularily sparse networks, there is no detectable improvement in PPV – see Figures 5(a)–(d).

In other words, Figures 4 and 5 compare the sensitivity and PPV of 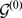 for a given threshold to the sensitivity and PPV of iPACOSE with the same threshold: iPACOSE has a real interest when there is room for improvement in 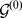
's PPV.

### Stability

In practical data analyses, the true network is almost always unknown, which makes the evaluation of graph inference methods so difficult on real data. For our particular application, we choose not to assess the performance of iPACOSE by comparing the obtained networks with interactions found in publicly available databases, but rather to evaluate its stability. A stable algorithm is robust against small perturbations of the dataset, see the work of Krämer *et al.* [7] or Varoquaux *et al.* [24] for an example in brain imaging. In our study, the considered datasets are split into 10 groups and GGMs are inferred based on datasets obtained by excluding each of the 10 groups successively. The 10 obtained networks are compared using Fleiss' 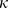, following the procedure described in Krämer *et al.* [7]. Fleiss' 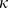 is originally designed to measure the degree of agreement between more than two raters. Each rater attributes a grade to an individual: in our case, a rater is a network inference method and a grade is 0 or 1, meaning that an interaction is considered as significant or not. The resulting statistic is always lower than 1 and, the closer the 10 networks, the closer it gets to 1. For a short description of this measure of agreement, see [25] (pp. 256–258).

**Figure 6:**
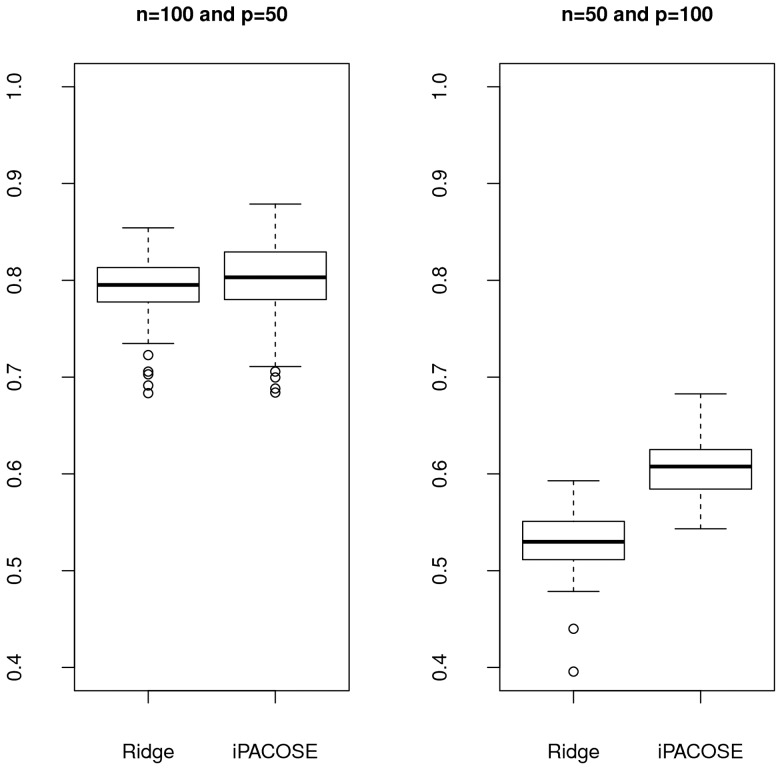
Measure of the stability with Fleiss' 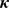 for the methods ridge.net and the Ridge version of iPACOSE. *LEFT FIGURE*: 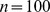 and 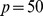. *RIGHT FIGURE*: 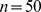 and 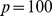. The regularization parameter of the ridge regression is determined analytically [26] for both methods.

**Figure 7:**
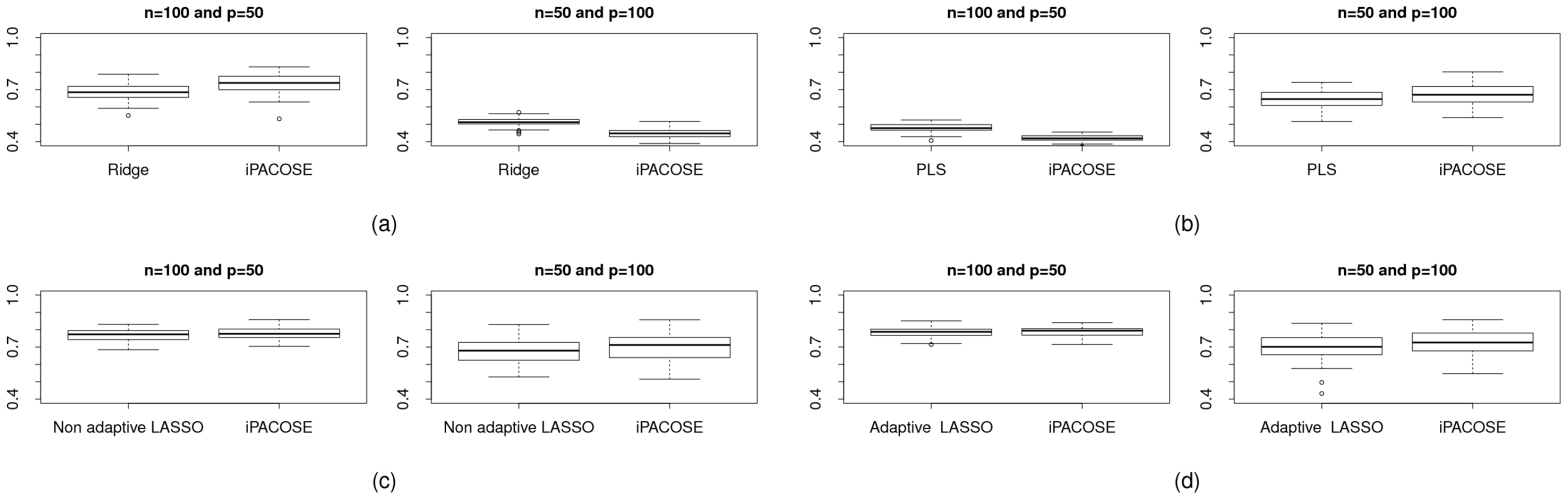
Measure of the stability with Fleiss' 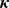 for (a) ridge.net and the Ridge version of iPACOSE, (b) pls.net and the PLS version of iPACOSE, (c) the non adaptive version of the method adalasso.net and the LASSO version of iPACOSE and (d) the adaptive version of the method adalasso.net and the adaptive LASSO version of iPACOSE. For each one of the couples of figures, the *LEFT FIGURE* corresponds to 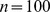 and 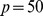 and the *RIGHT FIGURE* to 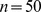 and 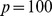. The regularization parameters of the Ridge, PLS, LASSO and adaptive LASSO regressions are determined analytically via a 5-fold cross-validation.

We first measure the stability of iPACOSE, and compare it to the stability of ridge.net on simulated data. According to Krämer *et al.* [7], ridge.net and the other methods presented in this paper do not show good stability performance. In order to stabilize the method, we replace the determination of the optimal ridge regularization parameter through a cross-validation approach by an analytic determination [26]. The results are shown on Figure 6. We observe that iPACOSE stabilizes the inference of the network. Figures 7(a), 7(b), 7(c) and 7(d) show the same type of stability results, except for the determination of the regularization parameters, which is done with a 5-fold cross validation. Stability is not improved with iPACOSE when it is low with the original inference method. However, when the stability is at a high level, it either remains at the same level or is improved.

### Application to a real dataset

This first very positive result still holds for the comparison of the stability of ridge.net and iPACOSE on a real dataset. For this application, we use the real data presented in [27] and further described and used in [28] consisting in 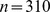 amino acid sequences on which were measured 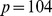 different physical properties. The regularization parameters are determined analytically [26] for both methods. Fleiss's 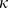 is computed on this dataset in a 10-fold fashion and is equal to 0.70 for the 10 networks obtained with ridge.net and to 0.85 for the 10 networks obtained with iPACOSE, which is an even higher improvement than in simulated data.

Replacing the 10-fold approach by a 5-fold does not essentially change the results (data not shown), which conforts us in the fact that our results are not depending too strongly on the number of parts the dataset is split into.

## Discussion

In this article, we presented PACOSE, a simple method to estimate a partial correlation matrix under the constraint that some known coefficients are null. We also presented iPACOSE, an original procedure to apply PACOSE iteratively within the estimation of independence graphs in combination with any GGM estimation method.

Our results on simulated data suggest that PACOSE's performance is very promising when the known graph describing the sparse structure of the partial correlation matrix is non-decomposable and 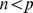. Since those two characteristics are met when dealing with biological data, our method is all the more interesting.

Having in mind the field of biological data as an application, we designed iPACOSE, an application of PACOSE to the estimation of independence graphs. iPACOSE is a method designed to improve the performance of the graph estimation algorithms based on the estimation of the partial correlation matrix. Results on simulated data show that iPACOSE manages to increase the positive predictive value of the inferred graphs while still showing good sensitivity. Moreover, results on simulated data and confirmed on real world data show that iPACOSE has very interesting stability properties. As a perspective of this work, iPACOSE would provide candidate interactions to work on more elaborate models, such as *e.g.* non linear ordinary differential equations applied to transcriptomic data [29] or used in cancer studies [30]. Such models would both help the discussion with the biologist or the phycisian by providing more elaborate interaction models between genes, and help in the design of “on the bench” experiments for the validation of the interactions found by iPACOSE.
